# Measure of synonymous codon usage diversity among genes in bacteria

**DOI:** 10.1186/1471-2105-10-167

**Published:** 2009-06-01

**Authors:** Haruo Suzuki, Rintaro Saito, Masaru Tomita

**Affiliations:** 1 Institute for Advanced Biosciences, Keio University, Tsuruoka, Yamagata, 997-0017, Japan; 2 Systems Biology Program, Graduate School of Media and Governance, Keio University, Fujisawa, Kanagawa, 252-8520, Japan

## Abstract

**Background:**

In many bacteria, intragenomic diversity in synonymous codon usage among genes has been reported. However, no quantitative attempt has been made to compare the diversity levels among different genomes. Here, we introduce a mean dissimilarity-based index (*D*mean) for quantifying the level of diversity in synonymous codon usage among all genes within a genome.

**Results:**

The application of *D*mean to 268 bacterial genomes shows that in bacteria with extremely biased genomic G+C compositions there is little diversity in synonymous codon usage among genes. Furthermore, our findings contradict previous reports. For example, a low level of diversity in codon usage among genes has been reported for *Helicobacter pylori*, but based on *D*mean, the diversity level of this species is higher than those of more than half of bacteria tested here. The discrepancies between our findings and previous reports are probably due to differences in the methods used for measuring codon usage diversity.

**Conclusion:**

We recommend that *D*mean be used to measure the diversity level of codon usage among genes. This measure can be applied to other compositional features such as amino acid usage and dinucleotide relative abundance as a genomic signature.

## Background

Most amino acids can be encoded by more than one codon (i.e., a triplet of nucleotides); such codons are described as being synonymous, and usually differ by one nucleotide in the third position. In most bacteria, alternative synonymous codons are not used with equal frequencies. Grantham *et al*. [1] showed that genes from same species often show similar patterns of codon usage, and proposed the 'genome hypothesis' that there exists a species-specific pattern of codon usage. Then, it was shown that in many organisms there are also considerable differences in codon usage among genes within a genome [2]. Previous analyses of codon usage diversity in bacteria have mostly focused on individual genomes, with no quantitative attempt to compare the diversity levels among different genomes. For comparative genomic analysis, it is desirable to quantify the level of codon usage diversity among genes in such a way that the estimates could be compared among genomes.

Different factors have been proposed to explain the preferential usage of a subset of synonymous codons, including biased mutation pressure (genome-wide mutational bias toward G/C or A/T) [3], difference in mutational bias between the leading and lagging strands of DNA replication (strand-specific mutational bias) [4,5], and natural selection for optimizing translation process (translational selection) [6]. Although the genome-wide mutational bias should act on the entire genome, the extent is stronger for the third positions of codons since the first two positions of codons are constrained by protein-coding requirements [7]. Thus, the mutational bias could be the cause of the preferential usage of either G/C- or A/T-ending codons. The strand-specific mutational bias could be the cause of the preferential usage of G/T- and C/A-ending codons in the leading and lagging strands, respectively [8,9]. The translational selection should act mainly on genes expressed at high levels in fast-growing bacteria [6]. The selection could be the cause of the preferential usage of translationally optimal codons, which are best recognized by the most abundant tRNA species in the cell [10,11]. It was reported that correlations of codon usage bias with gene expression level [6] and G+C content bias [12] are not ubiquitous. Thus, codon usage diversity within any genome could be the result of a balance among different evolutionary forces, and their relative contributions vary among different genomes.

Different methods have been used to examine codon usage diversity among genes [2,13-15]. Univariate statistics such as the 'effective number of codons' (ENC) [16] and G+C content at the third codon position (GC3) have been used to summarize codon usage of a gene. Representation of codon usage of a gene by a single statistic is essentially a reduction in information. GC3 estimates codon usage bias only toward either G/C- or A/T-ending codons. ENC estimates the degree of codon usage bias, but does not provide information about the types of preferred codons; thus two genes can exhibit same ENC values but prefer totally different codons. Multivariate analysis methods such as correspondence analysis (CA) have been used to construct axes accounting for the largest fractions of the total variation in codon usage among genes. In most genomes, the first two or three CA axes explain rather small amount of the total variation [13-15]. Carbone *et al*. [17] used the codon adaptation index (CAI) [18] as a universal measure of dominating codon usage bias. CA axis scores and CAI values derived from independent analyses cannot be compared. These limitations of previously used methods motivated us to consider alternative approach for measuring codon usage diversity.

In the present study, we introduce a mean dissimilarity-based index (*D*mean) for quantifying the level of diversity in synonymous codon usage among all genes within a genome. This index has been used to measure bacterial diversity [19,20]. The *D*mean values can be used to rank different genomes with respect to the overall codon usage diversity. The application of *D*mean to 268 bacterial genomes demonstrates that in bacteria with extremely biased genomic G+C compositions there is little diversity in synonymous codon usage among genes. Furthermore, our findings contradict the results of previous studies, and the reasons for the discrepancies are discussed.

## Results

### Synonymous codon usage diversity (*D*mean)

**Figure 1:**
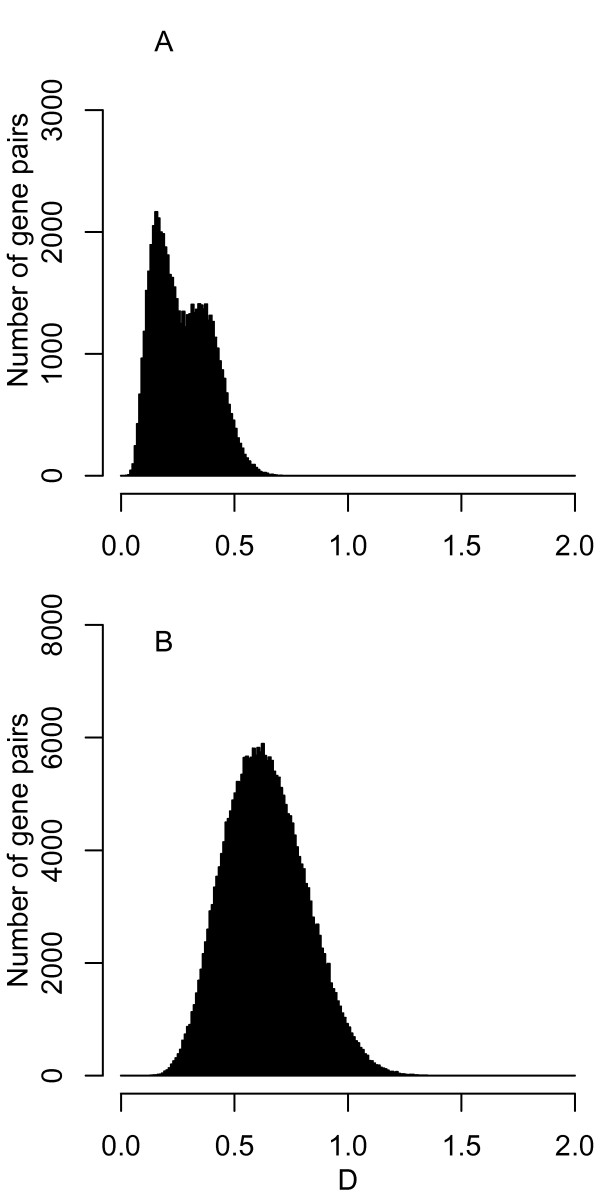
**Synonymous codon usage dissimilarity between genes**. Histograms showing the distribution of Pearson correlation distance (*D*) values between all pairs of all protein-coding genes within each of two genomes: *Borrelia burgdorferi *B31 (A) and *Treponema pallidum *Nichols (B).

To quantify the dissimilarity in synonymous codon usage between two genes, we calculated Pearson correlation distance (*D*). Figure 1 shows histograms generated by all pairwise *D *values among all protein-coding genes within each of two genomes: *Borrelia burgdorferi *B31 and *Treponema pallidum *Nichols as examples. In these two spirochaetes, there is a clear base composition skew between leading and lagging strands of replication [5]. The *D *values for *B. burgdorferi *exhibited a bimodal distribution with a left peak corresponding to within-strand dissimilarities and a right peak corresponding to between-strand dissimilarities (Figure 1A), whereas those for *T. pallidum *exhibited a monomodal distribution (Figure 1B). As a whole, the *D *values tended to be smaller in *B. burgdorferi *than in *T. pallidum*.

To quantify the level of diversity in synonymous codon usage among all genes within a genome, we calculated the mean distance (*D*mean) between all pairs of genes. The *D*mean value for *B. burgdorferi *(0.27) was smaller than that for *T. pallidum *(0.64), indicating that the diversity level was lower in *B. burgdorferi *than in *T. pallidum*. Among the 268 bacterial genomes tested here, the *D*mean values ranged from 0.09 to 0.70, with the median of 0.36 [see Additional file 1]. When focusing on previously studied genomes [13,14,21], the *D*mean values for *Bacillus subtilis *168 (0.60), *Escherichia coli *K12 MG1655 (0.47), *Helicobacter pylori *26695 (0.38), and *Haemophilus influenzae *Rd KW20 (0.37) were above the median, while those for *Pseudomonas aeruginosa *PAO1 (0.15) and *Streptomyces avermitilis *MA-4680 (0.14) were below the median. Thus, *D*mean values varied widely among bacteria.

### Relationship of *D*mean with genomic features

To investigate whether the level of intragenomic diversity in synonymous codon usage among genes is related to genomic features, we analyzed correlations of *D*mean with genomic G+C composition, replication strand skew, and tRNA gene number. The genomic G+C content (%GC) was expressed as 100 × (G+C)/(A+T+G+C). The strength of replication strand skew was quantified by the GC skew index (GCSI), which uses the power spectrum of Fourier transform of the graph of GC skew [the quantity (C-G)/(C+G)] and the Euclidean distance between the peaks [22]. Among the 268 bacterial genomes tested here, %GC, GCSI, and tRNA gene numbers varied from 22.5 to 74.9, 0.005 to 0.715, and 27 to 145, respectively [see Additional file 1].

**Figure 2:**
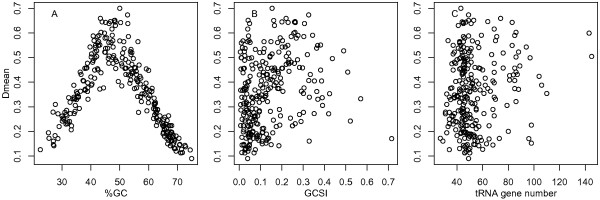
**Relationship of *D*mean with genomic features**. Scatter plots of *D*mean plotted against %GC (A), GCSI (B), and tRNA gene number (C) for 268 bacterial genomes.

Figure 2 shows scatter plots of the *D*mean values plotted against %GC, GCSI, and tRNA gene numbers for 268 bacterial genomes. The *D*mean values were nonlinearly correlated with %GC (Figure 2A). The highest *D*mean value (0.70) was found in *Prochlorococcus marinus *MIT 9303 (%GC = 50.0). The *D*mean values tended to be low in bacteria with extremely biased genomic G+C compositions (either G+C- or A+T-rich). Although these two types of genomes prefer different codons (either G/C- or A/T-ending codons), they can exhibit same *D*mean values. For example, *Wigglesworthia glossinidia *(endosymbiont of *Glossina brevipalpis*) and *Sorangium cellulosum *'*So ce 56*' had very different %GC (22.5 and 71.4, respectively) but exhibited same *D*mean values (0.13). In contrast to %GC (Figure 2A), GCSI (Figure 2B) and tRNA gene numbers (Figure 2C) were not clearly correlated with the *D*mean values.

### Comparison of *D*mean with previous methods

In previous studies, the extent of dispersal, e.g., range or standard deviation (SD), of univariate statistics such as the 'effective number of codons' (ENC) and G+C content at the third codon position (GC3) has been used to measure codon usage diversity among genes [13,14,21]. We compared *D*mean with SD of ENC and SD of GC3, designated as SD-ENC and SD-GC3, respectively.

**Figure 3:**
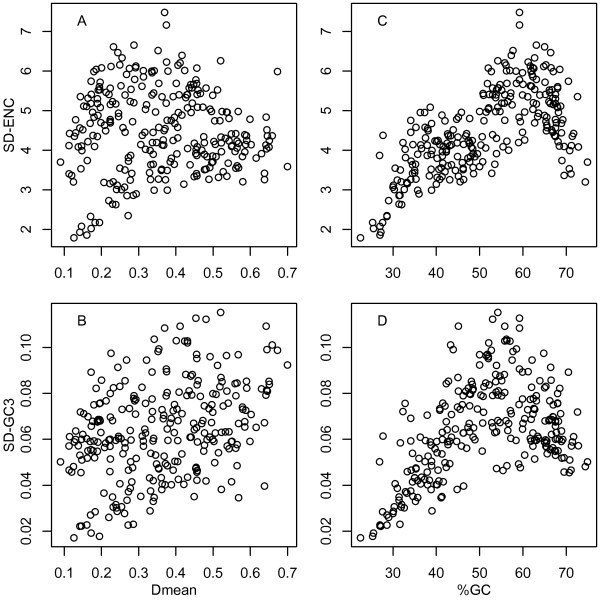
**Comparison of *D*mean with previous methods**. Scatter plots of SD-ENC and SD-GC3 against *D*mean (A and B) and %GC (C and D) for 268 bacterial genomes.

Figures 3A and 3B show scatter plots of SD-ENC and SD-GC3 plotted against *D*mean for the 268 bacterial genomes. The correlations of *D*mean with SD-ENC (Figure 3A) and SD-GC3 (Figure 3B) were unclear. The square of Pearson's product moment correlation coefficient of *D*mean with SD-ENC and SD-GC3 indicates that only 0.01% and 13.0% of the variance in *D*mean was explained by the variance in SD-ENC and SD-GC3, respectively.

Figures 3C and 3D show scatter plots of SD-ENC and SD-GC3 plotted against %GC for the 268 bacterial genomes. The nonlinear correlation with %GC was clearer when using *D*mean (Figure 2A) than when using SD-ENC (Figure 3C) and SD-GC3 (Figure 3D).

## Discussion

In many bacteria, intragenomic diversity in codon usage among genes has been reported [2,11]. However, no quantitative attempt has been made to compare the diversity levels among different genomes. Here, we used a mean distance (*D*mean) between all pairs of genes as a diversity index [20].

Different factors could contribute to codon usage diversity, including G+C composition, strand-specific mutational bias, and translational selection [23]. First, in bacteria with extremely biased genomic G+C compositions, synonymous codon usage could be dominated by strong genome-wide mutational biases [3,7]. The nonlinear correlation between *D*mean and %GC (Figure 2A) suggests that the biased mutational pressures homogenize codon usage throughout the genome. Such 'genome amelioration' is postulated to detect horizontally transferred genes based on unusual codon usage [24,25]. G+C composition could reflect not only mutational biases but also other factors such as chromosomal position [26], oxygen requirement [27], and energy cost and availability of nucleotides [28].

Second, in bacteria with clear base composition skews along the genome, synonymous codon usage could be subject to strong strand-specific mutational biases [8,9]. In *B. burgdorferi*, the *D*mean values for genes on the leading and lagging strands of replication were 0.19 and 0.20, respectively. The corresponding values in *T. pallidum *were 0.54 and 0.62. In these two spirochaetes, the *D*mean values for all genes (0.27 and 0.64, respectively) were larger than those for genes on each of the two replication strands, indicating that replication strand skew contributes to the overall codon usage diversity. The weak correlation between *D*mean and GCSI (Figure 2B) suggests that the strand-specific mutational biases contribute less to the overall codon usage diversity than the evolutionary forces that determine G+C composition.

Third, in bacteria with many tRNA genes, synonymous codon usage could be subject to strong translational selection [6]. The negligible correlation between *D*mean and tRNA gene numbers (Figure 2C) suggests that translational selection contributes little to the overall codon usage diversity. A possible explanation for this observation is that the number of highly expressed genes on which translational selection has been effective is a very small fraction of the genome.

The use of *D*mean led to the conclusions contrary to those drawn from previous studies. For example, a low level of heterogeneity in codon usage among genes has been reported for *H. pylori *genome in two independent analyses [14,17]. However, more than half of bacteria tested here had lower *D*mean values than the *D*mean value of *H. pylori *(0.38), indicating a moderate level of synonymous codon usage diversity in that genome. Also, clear and considerable heterogeneity in codon usage among genes has been reported for *P. aeruginosa *[13] and *S. avermitilis *[21], but their *D*mean values were very small (0.15 and 0.14, respectively), indicating a low level of synonymous codon usage diversity in these genomes. Previously used measures such as ENC (Figure 3A) and GC3 (Figure 3B) explain only a small percentage of the total variation in *D*mean. Furthermore, the nonlinear correlation between codon usage diversity and %GC was unclear when using ENC (Figure 3C) and GC3 (Figure 3D) instead of *D*mean (Figure 2A). Thus, the discrepancies between our findings and previous reports are probably due to differences in the methods used for measuring codon usage diversity.

## Conclusion

We recommend that *D*mean be used to measure the diversity level of codon usage among genes. This measure can be applied to other compositional features such as amino acid usage [29,30] and dinucleotide relative abundance as a genomic signature [31,32], and any groups of genes such as those encoding ribosomal proteins and aminoacyl-tRNA synthetases. The combined use of *D*mean and complementary methods [6,17,33-35] will improve our understanding of compositional diversity among genes.

## Methods

### Softwares

All analyses were implemented using the G-language Genome Analysis Environment version 1.8.3 [36,37] and the statistical software R version 2.6.1 [38].

### Sequences

Complete genome sequences of bacteria in GenBank format [39] were retrieved from the NCBI [40] FTP site. For each genus, only one representative strain was selected. The final data set included 268 different genomes [see Additional file 1]. Protein coding sequences containing letters other than A, C, G, or T, and those containing amino acids with residues less than their degree of codon degeneracy were discarded. From each coding sequence, methionine, tryptophan, and stop codons were excluded.

### Representation of synonymous codon usage of a gene

Synonymous codon usage of a coding sequence was represented by a vector, which consists of 59 variables (codons). The value of the *c*th codon for the *a*th amino acid (*x*_*ac*_) is defined as the ratio of the number of occurrences of a codon to the number of occurrences of the most abundant codon for the same amino acid [18].



where *n*_*ac *_is the number of occurrences of *c*th codon for the *a*th amino acid, and max(*n*_*ac*_) is the number of occurrences of the most frequently used synonymous codon for the *a*th amino acid. The *x*_*ac *_value is independent of three biases (i.e., gene length, amino acid composition, and codon degeneracy) which can mask effects of synonymous codon usage [35].

### Measure of synonymous codon usage diversity among genes

To quantify the dissimilarity in synonymous codon usage between two genes, we calculated Pearson correlation distance (*D*), defined as one minus Pearson's product moment correlation coefficient. Let *X*_*i *_and *X*_*j *_be the vectors consisting of 59 *x*_*ac *_values for the *i*th and *j*th genes, respectively. The *D *value between the *i*th and *j*th genes (*D*_*ij*_) was calculated as:



where cor(*X*_*i*_, *X*_*j*_) is the correlation coefficient of *X*_*i *_and *X*_*j*_. The correlation coefficient can vary from -1 (perfect negative correlation) through 0 (no correlation) to +1 (perfect positive correlation); thus the *D *value can vary from 0 (minimum dissimilarity) to 2 (maximum dissimilarity).

To quantify the level of diversity in synonymous codon usage among all genes, we calculated the mean distance (*D*mean) between all pairs of genes [20].



where *N *is the total number of genes. *D*mean can reach the minimum value of 0 when all genes prefer same synonymous codons for all amino acids.

## Abbreviations

A: adenine; T: thymine; G: guanine; C: cytosine; *D*: Pearson correlation distance; *D*mean: mean distance between all pairs of genes as a diversity index; %GC: genomic G+C content; GCSI: GC skew index; SD-ENC: standard deviation of the effective number of codons; SD-GC3: standard deviation of the G+C content at the third codon position.

## Authors' contributions

HS carried out the analysis and drafted the manuscript. RS and MT helped to draft the manuscript. All authors read and approved the final manuscript.

## Supplementary Material

Additional file 1 **Supplemental Table S1**. Statistics for the 268 bacterial genomes tested. Click here for file
